# Association Between Tea Drinking and Cognitive Disorders in Older Adults: A Meta-Analysis of Observational Studies

**DOI:** 10.3389/fnagi.2022.845053

**Published:** 2022-04-25

**Authors:** Mengyuan Shi, Limin Cao, Huiyuan Liu, Yuhan Zhou, Yuhong Zhao, Yang Xia

**Affiliations:** ^1^Department of Clinical Epidemiology, Shengjing Hospital of China Medical University, Shenyang, China; ^2^The Third Central Hospital of Tianjin, Tianjin, China

**Keywords:** tea drinking, older adults, meta-analysis, dose-response, congnitive disorders

## Abstract

**Introduction:**

Previous research has shown that tea drinking has a bearing on Cognitive Disorders, but the conclusions are inconsistent. The purpose of this research was to systematically assess the published evidence pertaining to tea drinking and the risk of cognitive disorders in older adults using a meta-analysis, and to concurrently evaluate the dose-response association.

**Design:**

A meta-analysis.

**Setting and Participants:**

We used the PubMed and Web of Science databases for a literature search until 30 May 2021. We initially retrieved 20,908 studies (14,884 from PubMed and 6,024 from the Web of Science), Thirty-six studies met the inclusion criteria (7 case-control, 16 cohort, and 13 cross-sectional studies), involved 224,980 participants.

**Methods:**

Pooled odd ratios (ORs) with their corresponding 95% confidence intervals (CIs) were used to evaluate the strength of the association under a fixed- or random-effect model according to heterogeneity test results.

**Results:**

The results showed that drinking tea was negatively associated with cognitive disorders (OR: 0.76, 95% CI: 0.70–0.82). Moreover, dose-response associations were found between tea drinking and cognitive disorders (1 time/day: OR, 0.81; 95% CI, 0.70–0.95; 1 cup/day: OR, 0.86; 95% CI, 0.78–0.94). In addition, subgroup analyses were performed according to study designs, study population, types of tea drinking, outcomes and methods used to assess outcomes. Most of the results in the subgroup analyses were consistent with the main results.

**Conclusion:**

The results of the present study provided abundant evidence that tea drinking is inversely proportional with the occurrence of cognitive disorders in older adults. A linear dose-response association between tea drinking and decreased prevalence of cognitive disorders was found.

## Summary

Cognitive disorders are a common neurodegenerative disease among older adults. The proportional and linear associations between tea drinking and cognitive disorders were studied by meta-analysis. A total of 36 studies (224,980 participants) were enrolled in the present study. Results demonstrated that tea drinking was positively associated with cognitive disorders.

## Introduction

Neurologic diseases impact the nervous system and include epilepsy, cerebrovascular disease (such as stroke), and neurodegenerative diseases (such as cognitive disorders) (Thangaraj et al., 2020). Cognitive disorders, also known as neurocognitive disorders, typically affect learning, memory, perceptual-motor function, language, attention, and problem solving (Robbins et al., 2019). Cognitive disorders mainly include delirium, mild cognitive impairment (MCI), and major neurocognitive disorder [such as dementia, Parkinson’s disease (PD), and Alzheimer’s disease (AD)] (Sachdev et al., 2014). After 65 years of age, the incidence of cognitive disorders increases sharply (Huang et al., 2009). Due to the increase in population aging worldwide, the disease burden of cognitive disorders is gradually increasing. For example, as a type of cognitive disorder, dementia affects 5–6% of people ≥ 60 years of age (Jia et al., 2020). The total number of dementia patients is expected to reach 82 million in 2030 and 152 million in 2050 (World Health Organization, 2020). Early intervention for cognitive disorders is especially effective, thus intervention can be used to help relieve the social and economic burden of AD and other types of cognitive disorders.

According to previous reports, cognitive disorders may be related to many factors, such as the aging process and lifestyle (Laudisio et al., 2008; Rabbitt et al., 2008a,b; Solfrizzi et al., 2008). Tea originated in China thousands of years ago and is widely enjoyed worldwide. In Asian countries, drinking green tea is a social custom, while Western countries prefer black tea (Cheng, 2006). It has been shown in experiments (*in vitro* studies, etc.) and animal studies that tea polyphenols may not only have effective neuroprotective activity, but may also help slow the progression of neurodegenerative diseases (Mandel and Youdim, 2004; Chen et al., 2018). *In vitro* and *in vivo* experimental studies have shown that tea and its components, such as catechin and theanine, have neuroprotective effects (Kim et al., 2009; Lee et al., 2009, 2013; Biasibetti et al., 2013). Some epidemiologic studies have shown that drinking tea can improve neuropsychiatric disorders (Gardner et al., 2007) and reduce the development of cognitive disorders (Feng et al., 2012). A previous study (Ide et al., 2014) concluded that drinking green tea ameliorates or delays cognitive disorders in older adults. Thus, green tea may delay the occurrence or development of cognitive disorders (Mandel et al., 2005, 2008; Song et al., 2012); however, other studies showed no association between tea drinking and the prevalence of cognitive disorders (Forster et al., 1995; Lindsay et al., 2002). These discrepant findings were the impetus for a meta-analysis to elucidate the association between tea drinking and cognitive disorders. Of note, existing meta-analyses involving tea drinking and cognitive disorders (Ran et al., 2021) have historically paid less attention to the association between subtypes of tea drinking and cognitive disorders.

To further understand the associations between tea drinking and cognitive disorders, we conducted subgroup analyses according to the types of tea drinking, study population, types of cognitive disorders, types of disease assessments, and study design. Moreover, the dose-response associations between tea drinking and cognitive disorders were further studied. The purpose of this research was to conduct a meta-analysis that provides a comprehensive conclusion on the associations between tea drinking and cognitive disorders in older adults, which is of clinical significance in efforts to prevent and treat cognitive disorders.

## Methods

### Literature Search

We used the PubMed and Web of Science databases for a literature search until 30 May 2021, with the following key words: (“tea” OR “oolong tea” OR “black tea” OR “subtypes of tea” OR “drinking tea” OR “beverage of tea” OR “tea consumption” OR “tea catechins consumption” OR “green tea” OR “tea intake” OR “intake of tea” OR “component in tea” OR “caffeine” OR “caffeine intake from tea” OR “tea polyphenol” OR “tea catechins” OR “tea extracts” OR “polyphenol” OR “catechins”) combined with (“Alzheimer’s disease” OR “cognitive impairment” OR “Alzheimer’s type” OR “cognitive decline” OR “dementia” OR “MCI” OR “mild neurocognitive disorder” OR “mild cognitive impairment” OR “cognitive impair*” OR “memory impair*” OR “Alzheimer’s disease” OR “dementia*”OR “cognitive dysfunction” OR “cognitive disorder*” OR “cognitive defect” OR “memory disorder*” OR “AD” OR “executive function” OR “Alzheimer*”). We also searched the selected literature in other correlative meta-analysis to gain the most integrated compilation of studies possible from the reported paper. In addition, in the event several studies originated from identical research, we chose the studies with the longest duration of follow-up and/or the largest sample size. We used Endnote 9.0 software for the search and management of the selected studies. The study selection process was performed following the Preferred Reporting Items for Systematic Review and Meta-analyses (PRISMA) statement (Page et al., 2021).

### Selection Criteria

The inclusion criteria for selected studies were as follows: (1) ≥ 60 of age; (2) selected studies included daily tea drinking (not tea extract) and cognitive disorder-related outcomes, such as AD, Parkinson’s disease (PD), and cognitive decline in older adults; and (3) studies that reported the odds ratios (ORs), hazard ratios (HRs), risk ratios (RRs), and corresponding 95% confidence intervals (CIs).

The animal studies, reviews, reports, and studies with unavailable data were excluded. If two or more studies shared the same population, we only selected the most recent study.

### Data Extraction and Quality Assessment

Two research assistants collected the following data from the literature: the first author’s last name; publication year; research country; study design; number of participants and cases; OR (95% CI); HR (95% CI); and RR (95% CI), as well as variables that were adjusted in the primary analysis. The Newcastle–Ottawa Scale (NOS) and the Agency for Healthcare Research and Quality (AHRQ) were used for quality assessment.

### Statistical Analysis

RRs and HRs are equal to ORs (Zhang and Yu, 1998; Li et al., 2012; Kim et al., 2015; Liu et al., 2017; Ran et al., 2021) when the prevalence of the outcome is low. An OR with a 95% CI determined the correlation between tea drinking and cognitive disorders. *I*^2^ was used to assess heterogeneity across studies. Based on the heterogeneity (*I*^2^ = 64.9%), a random effect model was selected. Influence analysis was also performed to determine whether an individual study affected the overall research results. Begg’s test was applied to assess publication bias. Subgroup analyses were carried out by disease type, population, type of study design, type of tea, and type of disease assessment.

A dose-response meta-analysis was performed using Stata software (version 16; Stata Corp., College Station, TX, United States), and the *glst* and *xblc* orders were applied to carry out the model estimation and draw linear or non-linear dose–response association maps.

## Results

### Study Selection

**FIGURE 1:**
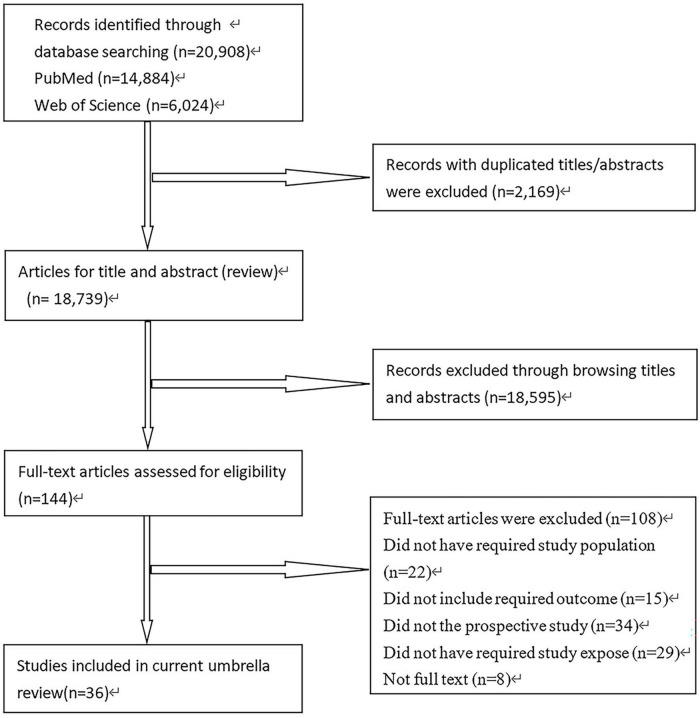
Study selection process for this meta-analysis.

The study selection process is shown in Figure 1. We preliminarily retrieved 20,908 studies (14,884 from PubMed and 6,024 from the Web of Science). First, we found 2,169 duplicate literature records through preliminary screening. Second, we screened the titles and abstracts, and 18,595 studies deviated significantly from the inclusion and exclusion criteria. Third, we conducted full-text screening, and 108 studies were excluded. Finally, 36 studies (7 case-control, 16 cohort, and 13 cross-sectional studies) met the inclusion criteria and were included for this meta-analysis with a total of 224,980 participants. Of which, 16 studies were from China, 6 were from Europe, 7 were from the North America, and 7 were from Japan.

### Study Characteristics

Table 1 summarizes the nature by country, study type, publication year, research design, etc. Supplementary Table 1 shows the distinguishing features of 36 studies (7 case-control, 13 cross-sectional, and 16 cohort studies). In the articles included in the study, different methods were used to measure tea drinking records, such as the Food Frequency Questionnaire (FFQ) and self-administered questionnaire. The great majority of studies were adjusted for latent confounding factors. We recorded the OR (95% CI) of cognitive disorders based on the highest and lowest tea drinking classifications.

**TABLE 1:** Summarizes the nature by country, study type, publication year, and research design.

Study	Study design	Country	Year	Number of participants	Outcome measurement method	Type	OR/RR	LCI	UCI
Forster, D. P.	Case-control study	United Kingdom	1995	218	Others	–	1.40	0.81	2.63
Paganini-Hill, A.	Case-control study	United States	2001	2,715	Others	–	1.21	0.86	1.70
Checkoway, H.	Case-control study	United States	2002	557	MMSE	–	0.40	0.20	0.90
Lindsay, J.	Case-control study	Canada	2002	4,088	3MSE	–	1.12	0.78	1.61
Kuriyama, S.	Cross-sectional study	Japan	2006	1,003	MMSE	–	0.62	0.43	0.89
Dai, Q.	Cohort study	United States	2006	1,589	others	–	1.70	0.67	4.33
Ritchie, K.	Cohort study	France	2007	7,017	MMSE	–	0.81	0.65	1.01
Vercambre, M. N.	Cohort study	France	2009	4,809	DECO score	Recent cognitive decline	0.96	0.78	1.19
					DECO score	Functional impairment	0.90	0.74	1.09
Nurk, E.	Cross-sectional study	Norway	2009	2,031	MMSE	–	0.68	0.49	0.93
Eskelinen, M. H.	Cross-sectional study	Finland	2009	1,409	MMSE	–	1.04	0.59	1.84
Huang, C. Q.	Cross-sectional study	China	2009	681	MMSE	Men	0.55	0.22	1.64
					MMSE	Women	0.96	0.38	2.45
Yao, Y. H.	Cohort study	China	2010	2,809	MMSE	–	0.57	0.42	0.77
Tanaka, K.	Case-control study	Japan	2011	617	others	–	0.59	0.35	1.00
Wu, M. S.	Cross-sectional study	China	2011	2,119	MMSE	–	0.99	0.75	1.30
Arab, L.	Cohort study	United States	2011	4,809	3MSE/IQCODE	Men	0.34	0.68	1.37
					3MSE/IQCODE	Women	0.62	0.18	1.41
Palacios, N.	Cohort study	United States	2012	112,122	Others	Men	0.72	0.40	1.27
					Others	Women	0.75	0.40	1.41
Chen, X., Y.	Case-control study	China	2012	5,691	MMSE	–	0.82	0.68	1.00
Yang, B., Q.	Case-control study	China	2014	720	MMSE	–	0.73	0.52	0.87
Wang, G.	Cohort study	China	2014	223	MMSE	–	0.48	0.21	1.11
Noguchi-Shinohara, M.	Cohort study	Japan	2014	490	CDR	Green tea	0.32	0.16	0.64
					CDR	Black tea	1.52	0.77	3.03
Shen, W.	Cross-sectional study	China	2015	9,375	MMSE	–	0.68	0.54	0.86
Zeng, Y.	Cohort study	China	2015	822	MMSE	–	0.59	0.35	1.01
Tomata, Y (a).	Cohort study	Japan	2016	13,645	The Kihon Checklist	–	0.73	0.61	0.87
Tomata, Y (b).	Cohort study	Japan	2016	14,402	The Kihon Checklist	–	0.79	0.70	0.88
Wang, T.	Cross-sectional study	China	2017	1,302	MMSE	–	0.72	0.49	1.07
An, R.	Cohort study	China	2017	4,749	MMSE	–	0.94	0.81	1.08
Gu, Y. J.	Cross-sectional study	China	2018	4,579	Others	–	0.74	0.57	0.98
Fischer, K.	Cohort study	Germany	2018	2,622	Others	–	0.94	0.86	1.02
Feng, L.	Cohort study	United States	2018	3,844	3MSE	–	1.19	0.81	1.75
Xu, H.	Cross-sectional study	China	2018	2,131	MoCA	Green tea for men	0.66	0.46	0.93
					MoCA	Green tea for women	0.82	0.58	1.16
					MoCA	Black tea for men	0.74	0.37	1.49
					MoCA	Black tea for women	0.52	0.24	1.12
					MoCA	Oolong tea for men	0.39	0.09	1.68
					MoCA	Oolong tea for women	0.60	0.13	2.72
Chuang, S. Y.	Cross-sectional study	China	2019	1,245	Others	–	0.61	0.44	0.85
Shirai, Y.	Cohort study	Japan	2020	1,305	MMSE	–	0.70	0.52	0.94
Matsuyama, S.	Cohort study	Japan	2020	2,923	Others	–	0.77	0.61	0.98
Qian, Yu−Xi	Cross-sectional study	China	2020	4,579	Others	–	0.66	0.48	0.90
Wang, Z.	Cross-sectional study	China	2020	625	MMSE	–	0.29	0.18	0.45
Huang, W. C.	Cross-sectional study	China	2021	1,115	MMSE	–	2.23	0.75	6.68

*CDR, Clinical Dementia Rating; DECO, the “Détérioration Cognitive Observeé” (observed cognitive deterioration) questionnaire; IQCODE, informant questionnaire on cognitive decline in the elderly; LCI, lower confidence limit; MMSE: Mini–Mental State Examination; OR, odds ratio; RR, relative risk; U.S., The United States; UK, The United Kingdom; UCI, upper confidence limit; 3MS, the modified Mini-Mental State.*

Supplementary Tables 2, 3 show the quality evaluation results of the included studies. According to the quality assessment by the NOS, the mean value for the 7 case-control and 16 cohort studies was 7.1 stars. In the current study, we considered a study awarded 5–9 stars as a medium- or high-quality study because the criteria for medium- or high-quality have not been established. All documents were of medium or high-quality with scores of 5–9. The cross-sectional study used the AHRQ scale for quality evaluation, including 11 items. Each item was rated as “yes” (1 point), and “no” or “unclear” (0 point); 0–3 points represented low-quality literature, 4–7 points represented medium-quality literature, and 8–11 points represented high-quality literature. All documents were of medium- or high-quality with scores of 5–8.

### Association Between Tea Drinking and Cognitive Function

**FIGURE 2:**
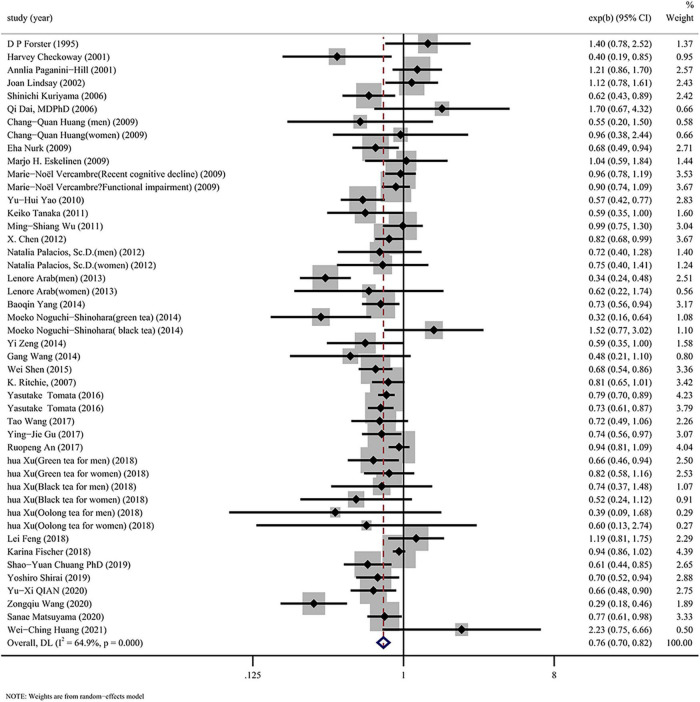
Overall pooled analysis of association between tea drinking and the cognitive disorders.

A notable heterogeneity (*I*^2^ = 64.9%) is shown in Figure 2. The forest map of the random effects model indicated that drinking tea was inversely proportional to cognitive disorders (OR: 0.76, 95% CI: 0.70–0.82).

### Subgroup Analyses

Table 2 shows the combined outcomes of each subgroup. According to the type of study design, we divided all the studies into three subgroups (Supplementary Figure 1). Among all three subgroups, cross-sectional (OR: 0.69, 95% CI: 0.61–0.79) and cohort studies (OR: 0.77, 95% CI: 0.69–0.87) indicated that tea drinking were inversely associated with cognitive disorders, while case-control studies showed no association. Among the population subgroups (Supplementary Figure 2), drinking tea remarkably lowered the risk of cognitive disorders in Chinese (OR: 0.70, 95% CI: 0.62–0.79), European (OR: 0.91, 95% CI: 0.83–0.99) and Japanese (OR: 0.73, 95% CI: 0.63–0.83) subgroups; however, no association existed in North American subgroup (OR: 0.83, 95% CI: 0.59–1.17). Among the subgroup analysis of types of cognitive disorders (Supplementary Figure 3), drinking tea remarkably lowered the risk of cognitive decline (OR: 0.71, 95% CI: 0.53–0.96), cognitive impairment (OR: 0.73, 95% CI: 0.65–0.82), and dementia (OR: 0.85, 95% CI: 0.76–0.95). Nevertheless, no correlation was demonstrated between tea drinking and PD (OR: 0.73, 95% CI: 0.50–1.08). Subgroup analysis of tea types (Supplementary Figure 4) showed that drinking green tea (OR: 0.75, 95% CI: 0.66–0.84) significantly reduced the risk of cognitive disorders, but not black tea (OR: 0.86, 95% CI: 0.58–1.28). Subgroup analysis was performed according to the type of disease assessment (Supplementary Figure 5). The Mini-Mental State Examination (MMSE) (OR: 0.70, 95% CI: 0.63–0.79), Montreal Cognitive Assessment (MoCA) (OR: 0.71, 95% CI: 0.59–0.86), Kihon Checklist (OR: 0.77, 95% CI: 0.70–0.85), Informant Questionnaire on Cognitive Decline in Older Adults (IQCODE) (OR: 0.37, 95% CI: 0.24–0.58), and other assessment types (OR: 0.79, 95% CI: 0.69–0.92) were associated with cognitive function, but the modified Mini-Mental State (3MSE) (OR: 0.74, 95% CI: 0.37–1.49), Clinical Dementia Rating (CDR) (OR: 0.51, 95% CI: 0.19–1.40), and Détérioration Cognitive Observeé (DECO) scores (OR: 0.93, 95% CI: 0.80–1.07) had no correlation between tea drinking and cognitive function.

**TABLE 2:** Combined results of subgroup analysis of tea drinking and cognitive function.

Subgroup analysis	Pooled OR (95%CI), *P*-value for the heterogeneity *Q*-test, *I*^2^ statistics (%), number of estimates in included studies (n)	
	*N*	Risk estimates of cognitive disorder
**Study design**		
Cohort	16	0.77 (0.69–0.87); *I*^2^ = 72.2%, *P* = 0.000
Cross-sectional	13	0.69 (0.61–0.79); *I*^2^ = 41.6%, *P* = 0.030
Case-control	7	0.87 (0.69–1.09); *I*^2^ = 63.1%, *P* = 0.012
**Type of tea drinking**		
Green tea	9	0.75 (0.66–0.84); *I*^2^ = 64.8%, *P* = 0.002
Black tea	4	0.86 (0.58–1.28); *I*^2^ = 55.6%, *P* = 0.061
**Study population**		
Chinese	16	0.70 (0.62–0.79); *I*^2^ = 55.3%, *P* = 0.001
Japanese	7	0.73 (0.63–0.83); *I*^2^ = 45.7%, *P* = 0.075
North American	7	0.83 (0.59–1.17); *I*^2^ = 79.8%, *P* = 0.000
European	6	0.91 (0.83–0.99); *I*^2^ = 16.9%, *P* = 0.301
**Type of cognitive disorders**		
Cognitive impairment	18	0.73 (0.65–0.82); *I*^2^ = 65.7%, *P* = 0.000
Dementia	14	0.85 (0.76–0.95); *I*^2^ = 61.9%, *P* = 0.001
PD	4	0.73 (0.50–1.08); *I*^2^ = 61.1%, *P* = 0.036
Cognitive decline	6	0.71 (0.53–0.96); *I*^2^ = 81.4%, *P* = 0.000
**Type of disease assessment**		
MMSE	20	0.70 (0.63–0.79); *I*^2^ = 55.6%, *P* = 0.000
3MSE	3	0.74 (0.37–1.49); *I*^2^ = 90.0%, *P* = 0.000
MoCA	2	0.71 (0.59–0.86); *I*^2^ = 0.0%, *P* = 0.904
The Kihon Checklist	3	0.77 (0.70–0.85); *I*^2^ = 0.0%, *P* = 0.464
CDR	2	0.51 (0.19–1.40); *I*^2^ = 87.9%, *P* = 0.000
IQCODE	1	0.37 (0.24–0.58); *I*^2^ = 14.8%, *P* = 0.279
DECO score	1	0.93 (0.80–1.07); *I*^2^ = 0.0%, *P* = 0.659
Others	14	0.79 (0.69–0.92); *I*^2^ = 62.9%, *P* = 0.001

*CDR, Clinical Dementia Rating; DECO, the “Détérioration Cognitive Observeé” (observed cognitive deterioration) questionnaire; IQCODE, informant questionnaire on cognitive decline in the elderly; MMSE: Mini–Mental State Examination; MoCA, Montreal Cognitive Assessment; PD, Parkinson’s disease; 3MSE, the modified Mini-Mental State.*

### Dose-Response Association Analysis

**FIGURE 3:**
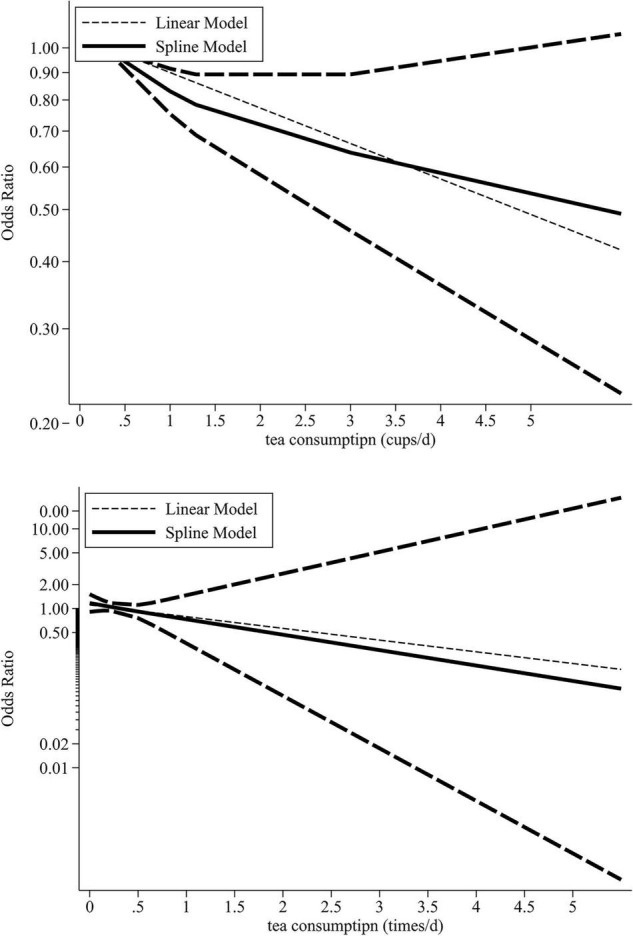
Dose-response association between tea drinking and risk of cognitive disorders.

Data for dose-response analysis were derived from 13 estimates of 11 separate studies. In addition to these studies, the remaining studies included two tea drinking datasets. Data from at least three dose levels are required for dose-response analysis, thus these data were excluded (Alexander et al., 2009). Tea drinking showed a negative linear association with cognitive disorders (*P* for linearity < 0.05; Figure 3). Therefore, drinking tea was a protective factor for cognitive disorders (1 time/day: OR, 0.81; 95% CI, 0.70–0.95; 1 cup/day: OR, 0.86; 95% CI, 0.78–0.94).

### Publication Bias

**FIGURE 4:**
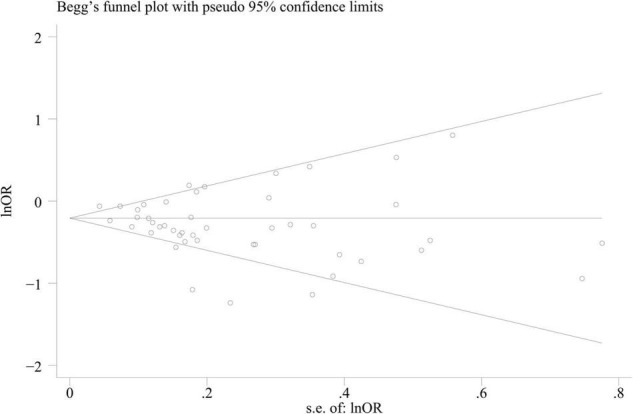
Begg’s funnel plot for identifying publication bias (*n* = 36).

Publication bias was evaluated based on the entire dataset. The *P*-value of the Begg’s test was 0.596, and the symmetric funnel plot is shown in Supplementary Figure 6. Based on the results of Begg’s test (*P* = 0.596; Figure 4), we did not detect any publication bias.

## Discussion

The results of the present study showed that tea drinking is negatively associated with cognitive disorders in older adults. The dose-response association confirmed that drinking tea markedly decreased the main outcome of cognitive disorders in older adults, especially green tea drinking.

Previous meta-analyses (Greenland, 1987; Ono et al., 2003; Li et al., 2012; Kim et al., 2015; Yamada et al., 2015; Liu et al., 2017; Ran et al., 2021) concluded that tea drinking is associated with cognitive disorders, which is consistent with the results in the present study. Caffeine is an important component in tea. Many experimental studies have shown the benefits of caffeine on cognitive function (Greenland, 1987). Other beneficial elements in tea, such as polyphenols, have anti-amyloidogenic effects (Alexander et al., 2009) and could be a key molecule for the development of preventives and therapeutics for cognitive disorders (Ono et al., 2003; Yamada et al., 2015).

Previous meta-analyses (Qi and Li, 2014; Liu et al., 2017) did not study the subgroup analysis association between tea drinking and cognitive disorders. In addition, some meta-analyses (Kim et al., 2015; Ma et al., 2016) did not examine the dose-response association between tea drinking and cognitive disorders. Most recently, a meta-analysis (Ran et al., 2021) showed that tea drinking has a linear association with morbidity and cognitive deficits. The results (Ran et al., 2021) showed that drinking 1 cup of tea per day leads to a 6% reduction in cognitive deficits morbidity, whereas 2 cups per day leads to an 11% decrease. No subgroup analysis was performed in this study, however (Ran et al., 2021). Moreover, the outcomes included in this meta-analysis (Ran et al., 2021) were MCI, AD, or dementia, and there was no outcome on PD, and cognitive decline. Another previous meta-analysis (Li et al., 2012) reported an association between tea drinking and PD. However, this meta-analysis only included PD as the outcome (Li et al., 2012). Overall, few previous meta-analysis studies have explored the associations between tea drinking and different kinds of cognitive disorders including MCI, dementia, AD, and PD. Thus, we conducted the present meta-analysis with the recently updated data among older adults as a multidimensional evaluation of the dose-response association between tea drinking and cognitive outcomes. Moreover, subgroup analyses were also conducted.

Cognitive disorders include a cluster of diseases (Battle, 2013; Gong et al., 2019), such as MCI, PD, AD, and dementia. The severity of these diseases differs (Roberts and Knopman, 2013; Masters et al., 2015; Tysnes and Storstein, 2017; World Health Organization, 2020). At the same time, the methods for measuring and defining diseases are different among studies (Ciesielska et al., 2016). Therefore, we conducted subgroup analyses according to types of cognitive disorders and disease assessment. In addition, different types of tea have different ingredients and different health effects (Mhatre et al., 2021). Different regions have different drinking habits and prefer different types of tea (Cabrera et al., 2006; Cheng, 2006; Chacko et al., 2010; Johnson et al., 2012). Therefore, we conducted subgroup analyses according to types of tea drinking and the study population. Finally, the study design (Mann, 2003) affects the reliability of the results. Thus, a subgroup analysis according to different study designs was performed.

The subgroup analysis showed that drinking tea was associated with cognitive impairment, cognitive decline, and dementia, but no apparent association was found in the outcome of PD. This finding may be partially attributed to the limited evidence between tea drinking and PD in the studies we included. The subgroup analysis showed that drinking tea may be associated with cognitive disorders among Chinese, Japanese, and European subgroups, but no such association was found in the North American subgroup. We speculate that the main reason for this finding may be related to the different kinds of popular tea in different areas. For example, North Americans prefer black tea to green tea. The nutritional and functional components of black and green teas are not quite the same. For example, the levels of catechin (ECGC) are highest in green tea, followed by black tea (Lin et al., 2003). Moreover, green tea contains ascorbic acid and a high intake of ascorbic acid is related to a reduced risk of AD (Engelhart et al., 2002); however, black tea does not contain ascorbic acid (Noguchi-Shinohara et al., 2014). This finding was also confirmed by our subgroup analysis which showed green tea, but not black tea, was negatively associated with cognitive disorders. Subgroup analysis also showed that the type of disease assessment can have an impact on the outcome. Future studies should take into consideration the type of assessment used when assessing different cognitive disorders.

The advantages of this meta-analysis were as follows: First, the symmetric distribution included in the study enhanced the reliability of the statistical analysis. Second, the literature included in our meta-analysis was of medium-to-high quality, which made the result more stable. Third, this meta-analysis included as much literature as possible (Kim et al., 2015), which not only expanded the sample population, but also added the subgroup analysis, which enhanced the evidence strength of the study. Finally, the dose-response analysis improved the quality and intensity of our findings.

The present meta-analysis had some potential limitations. First, we detected slight heterogeneity in the study, which could be interpreted by differences in population source, study design, tea drinking type, cognitive disorders detection, and analysis strategies. Second, some important confounding factors were not adjusted in the initial study, such as lifestyle (diet and interest), and some related conditions (urinary disease and dyslipidemia). Therefore, we could not rule out confounders, such as accidental, residual, or unmeasured factors, which may have caused our results to be biased. Third, in most of the studies included, the data of tea drinking came from self-administered questionnaires, which inevitably lead to misclassification. Fourth, although the result was derived from a few studies, the studies were not comprehensive in terms of tea subtype, study population, study type, assessment type, and disease type, which may have led to unstable or limited secondary analysis results. Finally, we treated HR/RR equal to OR according to the published studies (Li et al., 2012; Ran et al., 2021) which may have affected the results. A previous study (Bigby, 2000) suggested that using the OR as an approximation of the RR produces progressively larger errors as the outcome rate rises above 1%.

## Conclusion

In conclusion, a total of 36 independent observational studies were included and supported that tea drinking is inversely proportional and linear associated with the occurrence of cognitive disorders in older adults. Future randomized controlled trials of tea (not tea extract) are needed to confirm our results.

## Data Availability Statement

The original contributions presented in the study are included in the article/Supplementary Material, further inquiries can be directed to the corresponding author/s.

## Author Contributions

MS: conceptualization, formal analysis, visualization, and writing-original draft. LC: writing-review and editing, and supervision. HL, YZho, and YZha: writing-review and editing. YX: conceptualization, resources, writing-review and editing, supervision, and funding acquisition. All authors contributed to the article and approved the submitted version.

## Conflict of Interest

The authors declare that the research was conducted in the absence of any commercial or financial relationships that could be construed as a potential conflict of interest.

## Publisher’s Note

All claims expressed in this article are solely those of the authors and do not necessarily represent those of their affiliated organizations, or those of the publisher, the editors and the reviewers. Any product that may be evaluated in this article, or claim that may be made by its manufacturer, is not guaranteed or endorsed by the publisher.

## References

[B1] Alexander D. D. Cushing C. A. Lowe K. A. Sceurman B. Roberts M. A. (2009). Meta-analysis of animal fat or animal protein intake and colorectal cancer. *Am. J. Clin. Nutr.* 89 1402–1409. 10.3945/ajcn.2008.26838 19261724

[B2] Battle D. E. (2013). Diagnostic and statistical manual of mental disorders (DSM). *Codas* 25 191–192. 24413388 10.1590/s2317-17822013000200017

[B3] Biasibetti R. Tramontina A. C. Costa A. P. Dutra M. F. Quincozes-Santos A. Nardin P. (2013). Green tea (-)epigallocatechin-3-gallate reverses oxidative stress and reduces acetylcholinesterase activity in a streptozotocin-induced model of dementia. *Behav. Brain Res.* 236 186–193. 10.1016/j.bbr.2012.08.039 22964138

[B4] Bigby M. (2000). Odds ratios and relative risks. *Arch. Dermatol.* 136 770–771. 10871942 10.1001/archderm.136.6.770

[B5] Cabrera C. Artacho R. Giménez R. (2006). Beneficial effects of green tea–a review. *J. Am. Coll. Nutr.* 25 79–99. 10.1080/07315724.2006.10719518 16582024

[B6] Chacko S. M. Thambi P. T. Kuttan R. Nishigaki I. (2010). Beneficial effects of green tea: a literature review. *Chin. Med.* 5:13. 10.1186/1749-8546-5-13 20370896 PMC2855614

[B7] Chen S. Q. Wang Z. S. Ma Y. X. Zhang W. Lu J. L. Liang Y. R. (2018). Neuroprotective effects and mechanisms of tea bioactive components in neurodegenerative diseases. *Molecules* 23:512. 10.3390/molecules23030512 29495349 PMC6017384

[B8] Cheng T. O. (2006). All teas are not created equal: the Chinese green tea and cardiovascular health. *Int. J. Cardiol.* 108 301–308. 10.1016/j.ijcard.2005.05.038 15978686

[B9] Ciesielska N. Sokołowski R. Mazur E. Podhorecka M. Polak-Szabela A. Kędziora-Kornatowska K. (2016). Is the montreal cognitive assessment (MoCA) test better suited than the mini-mental state examination (MMSE) in mild cognitive impairment (MCI) detection among people aged over 60? meta-analysis. *Psychiatr. Pol.* 50 1039–1052. 10.12740/PP/45368 27992895

[B10] Engelhart M. J. Geerlings M. I. Ruitenberg A. van Swieten J. C. Hofman A. Witteman J. C. (2002). Dietary intake of antioxidants and risk of Alzheimer disease. *JAMA* 287 3223–3229. 10.1001/jama.287.24.3223 12076218

[B11] Feng L. Li J. Ng T. P. Lee T. S. Kua E. H. Zeng Y. (2012). Tea drinking and cognitive function in oldest-old Chinese. *J. Nutr. Health Aging* 16 754–758. 10.1007/s12603-012-0077-1 23131816 PMC3675265

[B12] Forster D. P. Newens A. J. Kay D. W. Edwardson J. A. (1995). Risk factors in clinically diagnosed presenile dementia of the Alzheimer type: a case-control study in northern England. *J. Epidemiol. Community Health* 49 253–258. 10.1136/jech.49.3.253 7629459 PMC1060793

[B13] Gardner E. J. Ruxton C. H. Leeds A. R. (2007). Black tea–helpful or harmful? A review of the evidence. *Eur. J. Clin. Nutr.* 61 3–18. 10.1038/sj.ejcn.1602489 16855537

[B14] Gong Z. Wang S. Chen W. (2019). Current situation and trend of rehabilitation for cognitive impairment. *West China Med. J.* 34 487–493.

[B15] Greenland S. (1987). Quantitative methods in the review of epidemiologic literature. *Epidemiol. Rev.* 9 1–30. 10.1093/oxfordjournals.epirev.a036298 3678409

[B16] Huang C. Q. Dong B. R. Zhang Y. L. Wu H. M. Liu Q. X. (2009). Association of cognitive impairment with smoking, alcohol consumption, tea consumption, and exercise among Chinese nonagenarians/centenarians. *Cogn. Behav. Neurol.* 22 190–196. 10.1097/WNN.0b013e3181b2790b 19741330

[B17] Ide K. Yamada H. Takuma N. Park M. Wakamiya N. Nakase J. (2014). Green tea consumption affects cognitive dysfunction in the elderly: a pilot study. *Nutrients* 6 4032–4042. 10.3390/nu6104032 25268837 PMC4210905

[B18] Jia L. Du Y. Chu L. Zhang Z. Li F. Lyu D. (2020). Prevalence, risk factors, and management of dementia and mild cognitive impairment in adults aged 60 years or older in China: a cross-sectional study. *Lancet Public Health* 5 e661–e671. 10.1016/S2468-2667(20)30185-7 33271079

[B19] Johnson R. Bryant S. Huntley A. L. (2012). Green tea and green tea catechin extracts: an overview of the clinical evidence. *Maturitas* 73 280–287. 10.1016/j.maturitas.2012.08.008 22986087

[B20] Kim T. I. Lee Y. K. Park S. G. Choi I. S. Ban J. O. Park H. K. (2009). l-Theanine, an amino acid in green tea, attenuates beta-amyloid-induced cognitive dysfunction and neurotoxicity: reduction in oxidative damage and inactivation of ERK/p38 kinase and NF-kappaB pathways. *Free Radic. Biol. Med.* 47 1601–1610. 10.1016/j.freeradbiomed.2009.09.008 19766184

[B21] Kim Y. S. Kwak S. M. Myung S. K. (2015). Caffeine intake from coffee or tea and cognitive disorders: a meta-analysis of observational studies. *Neuroepidemiology* 44 51–63. 10.1159/000371710 25721193

[B22] Laudisio A. Marzetti E. Pagano F. Cocchi A. Franceshi C. Bernabei R. (2008). Association of metabolic syndrome with cognitive function: the role of sex and age. *Clin. Nutr.* 27 747–754. 10.1016/j.clnu.2008.07.001 18715681

[B23] Lee J. W. Lee Y. K. Ban J. O. Ha T. Y. Yun Y. P. Han S. B. (2009). Green tea (-)-epigallocatechin-3-gallate inhibits beta-amyloid-induced cognitive dysfunction through modification of secretase activity via inhibition of ERK and NF-kappaB pathways in mice. *J. Nutr.* 139 1987–1993. 10.3945/jn.109.109785 19656855

[B24] Lee Y. J. Choi D. Y. Yun Y. P. Han S. B. Oh K. W. Hong J. (2013). Epigallocatechin-3-gallate prevents systemic inflammation-induced memory deficiency and amyloidogenesis via its anti-neuroinflammatory properties. *J. Nutr. Biochem.* 24 298–310. 10.1016/j.jnutbio.2012.06.011 22959056

[B25] Li F. J. Ji H. F. Shen L. (2012). A meta-analysis of tea drinking and risk of Parkinson’s disease. *ScientificWorldJournal* 2012:923464. 10.1100/2012/923464 22448141 PMC3289976

[B26] Lin Y. S. Tsai Y. J. Tsay J. S. Lin J. K. (2003). Factors affecting the levels of tea polyphenols and caffeine in tea leaves. *J. Agric Food Chem.* 51 1864–1873. 10.1021/jf021066b 12643643

[B27] Lindsay J. Laurin D. Verreault R. Hébert R. Helliwell B. Hill G. B. (2002). Risk factors for Alzheimer’s disease: a prospective analysis from the Canadian study of health and aging. *Am. J. Epidemiol.* 156 445–453. 10.1093/aje/kwf074 12196314

[B28] Liu X. Du X. Han G. Gao W. (2017). Association between tea consumption and risk of cognitive disorders: a dose-response meta-analysis of observational studies. *Oncotarget* 8 43306–43321. 10.18632/oncotarget.17429 28496007 PMC5522147

[B29] Ma Q. P. Huang C. Cui Q. Y. Yang D. J. Sun K. Chen X. (2016). Meta-analysis of the association between tea intake and the risk of cognitive disorders. *PLoS One* 11:e0165861. 10.1371/journal.pone.016586 PMC5100989 27824892

[B30] Mandel S. Youdim M. B. (2004). Catechin polyphenols: neurodegeneration and neuroprotection in neurodegenerative diseases. *Free Radic. Biol. Med.* 37 304–317. 10.1016/j.freeradbiomed.2004.04.012 15223064

[B31] Mandel S. A. Amit T. Weinreb O. Reznichenko L. Youdim M. B. (2008). Simultaneous manipulation of multiple brain targets by green tea catechins: a potential neuroprotective strategy for Alzheimer and Parkinson diseases. *CNS Neurosci. Ther.* 14 352–365. 10.1111/j.1755-5949.2008.00060.x 19040558 PMC6493995

[B32] Mandel S. A. Avramovich-Tirosh Y. Reznichenko L. Zheng H. Weinreb O. Amit T. (2005). Multifunctional activities of green tea catechins in neuroprotection. Modulation of cell survival genes, iron-dependent oxidative stress and PKC signaling pathway. *Neurosignals* 14 46–60. 10.1159/000085385 15956814

[B33] Mann C. J. (2003). Observational research methods. Research design II: cohort, cross sectional, and case-control studies. *Emerg. Med. J.* 20 54–60. 10.1136/emj.20.1.54 12533370 PMC1726024

[B34] Masters C. L. Bateman R. Blennow K. Rowe C. C. Sperling R. A. Cummings J. L. (2015). Alzheimer’s disease. *Nat. Rev. Dis. Primers* 1:15056. 10.1038/nrdp.2015.56 27188934

[B35] Mhatre S. Srivastava T. Naik S. Patravale V. (2021). Antiviral activity of green tea and black tea polyphenols in prophylaxis and treatment of COVID-19: a review. *Phytomedicine* 85:153286. 10.1016/j.phymed.2020.153286 32741697 PMC7367004

[B36] Noguchi-Shinohara M. Yuki S. Dohmoto C. Ikeda Y. Samuraki M. Iwasa K. (2014). Consumption of green tea, but not black tea or coffee, is associated with reduced risk of cognitive decline. *PLoS One* 9:e96013. 10.1371/journal.pone.0096013 24828424 PMC4020750

[B37] Ono K. Yoshiike Y. Takashima A. Hasegawa K. Naiki H. Yamada M. (2003). Potent anti-amyloidogenic and fibril-destabilizing effects of polyphenols in vitro: implications for the prevention and therapeutics of Alzheimer’s disease. *J. Neurochem.* 87 172–181. 10.1046/j.1471-4159.2003.01976.x 12969264

[B38] Page M. J. McKenzie J. E. Bossuyt P. M. Boutron I. Hoffmann T. C. Mulrow C. D. (2021). The PRISMA 2020 statement: an updated guideline for reporting systematic reviews. *BMJ* 372:n71. 10.1136/bmj.n71 PMC8005924 33782057

[B39] Qi H. Li S. (2014). Dose-response meta-analysis on coffee, tea and caffeine consumption with risk of Parkinson’s disease. *Geriatr. Gerontol. Int.* 14 430–439. 10.1111/ggi.12123 23879665

[B40] Rabbitt P. Ibrahim S. Lunn M. Scott M. Thacker N. Hutchinson C. (2008a). Age-associated losses of brain volume predict longitudinal cognitive declines over 8 to 20 years. *Neuropsychology* 22 3–9. 10.1037/0894-4105.22.1.3 18211150

[B41] Rabbitt P. Lunn M. Ibrahim S. Cobain M. McInnes L. (2008b). Unhappiness, health and cognitive ability in old age. *Psychol. Med.* 38 229–236. 10.1017/S0033291707002139 17988418

[B42] Ran L. S. Liu W. H. Fang Y. Y. Xu S. B. Li J. Luo X. (2021). Alcohol, coffee and tea intake and the risk of cognitive deficits: a dose-response meta-analysis. *Epidemiol. Psychiatr. Sci.* 30:e13. 10.1017/S2045796020001183 33568254 PMC8061189

[B43] Robbins R. N. Scott T. Joska J. A. Gouse H. (2019). Impact of urbanization on cognitive disorders. *Curr. Opin. Psychiatry* 32 210–217. 10.1097/YCO.0000000000000490 30695001 PMC6438716

[B44] Roberts R. Knopman D. S. (2013). Classification and epidemiology of MCI. *Clin. Geriatr. Med.* 29 753–772. 10.1016/j.cger.2013.07.003 24094295 PMC3821397

[B45] Sachdev P. S. Blacker D. Blazer D. G. Ganguli M. Jeste D. V. Paulsen J. S. (2014). Classifying neurocognitive disorders: the DSM-5 approach. *Nat. Rev. Neurol.* 10 634–642. 10.1038/nrneurol.2014.181 25266297

[B46] SolfrizziV. CapursoC. D’IntronoA. ColaciccoA. M. FrisardiV. SantamatoA. (2008). Dietary fatty acids, age-related cognitive decline, and mild cognitive impairment. *J. Nutr. Health Aging* 12 382–386. 10.1007/BF02982670 18548175

[B47] SongJ. XuH. LiuF. FengL. (2012). Tea and cognitive health in late life: current evidence and future directions. *J. Nutr. Health Aging* 16 31–34. 10.1007/s12603-011-0139-9 22237999

[B48] Thangaraj A. Sil S. Tripathi A. Chivero E. T. Periyasamy P. Buch S. (2020). Targeting endoplasmic reticulum stress and autophagy as therapeutic approaches for neurological diseases. *Int. Rev. Cell. Mol. Biol.* 350 285–325. 10.1016/bs.ircmb.2019.11.001 32138902

[B49] Tysnes O. B. Storstein A. (2017). Epidemiology of Parkinson’s disease. *J. Neural Transm. (Vienna)* 124 901–905. 28150045 10.1007/s00702-017-1686-y

[B50] World Health Organization [WHO] (2020). *Dementia: A Public Health Priority.* Geneva: WHO.

[B51] Yamada M. Ono K. Hamaguchi T. Noguchi-Shinohara M. (2015). Natural phenolic compounds as therapeutic and preventive agents for cerebral amyloidosis. *Adv. Exp. Med. Biol.* 863 79–94. 10.1007/978-3-319-18365-7_4 26092627

[B52] Zhang J. Yu K. F. (1998). What’s the relative risk? A method of correcting the odds ratio in cohort studies of common outcomes. *JAMA* 280 1690–1691. 9832001 10.1001/jama.280.19.1690

