# Supplementary material for: Association Between Tea Drinking and Cognitive Disorders in Older Adults: A Meta-Analysis of Observational Studies
Source: Front Aging Neurosci. 2022 Apr 25;14:845053. doi: 10.3389/fnagi.2022.845053 (PMC9083466; doi:10.3389/fnagi.2022.845053)
Supplement: Supplementary file 1 [file Data_Sheet_1.docx]

**Table S1. Characteristics of the included studies in this meta-analysis.**

| Author Year | Country | Study Design | Number of participants | Age (year) | Follow-up Duration (years) | Exposure variable | Disease Type | Results | Adjusted Covariate |
| --- | --- | --- | --- | --- | --- | --- | --- | --- | --- |
| Forster, D. P., et al. 1995 | UK | case-control study | 218 | ≥ 65 | - | tea | AD | OR (95% CI): 1.40 (0.81–2.63) | —— |
| Paganini-Hill, A. et al 2001 | U.S. | case-control study | 2,715 (395/2,320) | the baseline survey: 75 ± 6.1y (case)/75 ± 6.0y (control); death or December 31: 85 ± 5.9y (case)/ 85 ± 5.8y (control) | 12 | tea | PD | OR (95% CI): 1.21 (0.86–1.70) | sex, birth date (±1 year), vital status, and, if dead, death date (±1 year). |
| Checkoway, H., et al. 2002 | U.S. | case-control study | 557 (210/347) | 71 (mean age) | 8 | tea | PD | OR (95% CI): 0.4 (0.2–0.9) | —— |
| Lindsay, J., et al. 2002 | Canada | case-control study | 4,088 (194/3,894) | ≥ 65 | 5 | tea | AD/Dementia | OR (95% CI) :1.12 (0.78–1.61) | —— |
| Kuriyama, S., et al. 2006 | Japan | cross-sectional study | 1,003 | ≥70 | - | green tea | Cognitive Impairment | OR (95% CI): 0.62 (0.43–0.89) | age, sex, and duration of education, visiting friends, smoking, alcohol use, physical activity, history of chronic medical conditions such as stroke or myocardial infarction, regular intake of supplements, medication, self-rated health. |
| Dai, Q., et al. 2006 | U.S. | cohort study | 1,589 | ≥65 | 9 | tea | AD | HR (95% CI): 1.70 (0.67–4.33) | —— |
| Ritchie, K., et al. 2007 | France | cohort study | 7,017 | ≥65 | 3.47 | tea | Dementia | OR (95% CI): 0.81 (0.65–1.02) | sex, educational level, and center, and taking age. |
| Vercambre, M. N., et al. 2009 | France | cohort study | 4,809 | ≥ 60 | 13 | tea | Cognitive Decline | OR (95% CI) [Recent cognitive decline (DECO score＞33) :0.96 (0.78–1.19); Functional impairment (4-IADL score＞0): 0.90 (0.74–1.09)] | —— |
| Nurk, E., et al. 2009 | Norway | cross-sectional study | 2,031 | 70–74 | - | tea | Poor Cognitive Performance | OR (95% CI): 0.68 (0.49–0.93) | sex, education, vitamin supplement use, smoking status, history of CVD, diabetes, and total energy intake. |
| Eskelinen, M. H., et al. 2009 | Finland | cross-sectional study | 1,409 (543/875) | 65–79 | - | tea | Dementia/AD | OR (95% CI): 1.04 (0.59–1.84) | midlife age, sex, education, follow-up time, community of residence, midlife smoking, SBP, DBP, serum total cholesterol, BMI, and physical activity. |
| Huang, C. Q., et al. 2009 | China | cross-sectional study | 681 | 90–108 | - | tea | Cognitive Impairment | OR (95% CI): [men: 0.917 (0.344–2.449); women: 0.862 (0.265–0.907)] | age, educational levels, sleep time one day, satisfaction of sleep, sleep at noon, body mass index (BMI, kg/m2), religion (yes or no), and temperament and depression scores. |
| Yao, Y. H., et al. 2010 | China | cohort study | 2,809 | 60–92 | - | tea | Cognitive Impairment | OR (95% CI): 0.57 (0.42–0.77) | age, gender, educational level, marriage, alcohol consumption, smoking, drinking tea, physical activities and preferring a no salty diet. |
| Tanaka, K., et al. 2011 | Japan | case-control study | 617 (249/368) | ≥60 | 2 | black tea, Japanese and Chinese teas | PD | OR (95% CI): 0.59 (0.35–0.995) | sex, age, region of residence, educational level, pack-years of smoking, body mass index, glycemic index, and intake of cholesterol, vitamin E, b-carotene, vitamin B6, alcohol, and iron were used as confounding variables. |
| Wu, M. S., et al. 2011 | China | cross-sectional study | 2,119 | ≥ 65 | - | tea | Cognitive Impairment | OR (95% CI): 0.99 (0.75–1.30) | age, gender, education level, marital status, and social support. |
| Arab, L., et al. 2011 | U.S. | cohort study | 4,809 | ≥65 | 6 | tea | Cognitive Decline | OR (95% CI): [men: 0.34 (0.68–1.37); women: 0.62 (0.18–1.41)] | age, race, educational attainment, field center, history of stroke, history of CHD, diabetes, hypertension, current smoking, depression score, and APOEε4. |
| Palacios, N., et al. 2012 | U.S. | cohort study | 112,122 | 71 (man) and 69 (woman) | 10 | tea | PD | RR (95% CI): [men: 0.72 (0.40–1.27); women: 0.75 (0.40–1.41)] | —— |
| Chen, X., Y., et al. 2012 | China | case-control study | 5,691 (1,389/4,302) | ≥65 | 3 | tea | Cognitive Decline | OR (95% CI): 0.82 (0.68–1.00) | age, gender, marital status, financial status, residential area, BMI, hypertension, diabetes, smoking, alcohol, tea drinking, and exercise habits. |
| Yang, B., Q., et al. 2014 | China | case-control study | 720 (240/480) | ≥ 65 | - | tea | AD | OR (95% CI): 0.725 (0.523–0.867) | age, sex, smoking, drinking, living conditions, social activities, physical exercise, leisure activities, diet, tea drinking. |
| Wang, G., et al. 2014 | China | cohort study | 223 | ≥ 65 | 2 | green tea | Cognitive Decline | OR (95% CI): 0.48 (0.21–1.11) | age, non- Chinese speaking back ground and education, and a formal diagnosis of dementia. |
| Noguchi-Shinohara, M., et al. 2014 | Japan | cohort study | 490 | ＞60 | 1 | green tea, black tea | Dementia and MCI | OR (95% CI): [green tea: 0.32 (0.16–0.64); black tea 1.52 (0.77–3.03)] | age, sex, history of hypertension, diabetes mellitus, hyperlipidemia, education, APOE E4 carrier status, alcohol drinking, smoking, physical activities and/or hobbies, and coffee and black tea consumption. |
| Shen, W., et al. 2015 | China | cross-sectional study | 9,375 | ≥ 60 | - | tea | Cognitive Impairment | OR (95% CI): [ CCM: 0.49 (0.36–0.66); MMSE score <24: 0.68 (0.54–0.86)] | demographic characteristics (age, gender, race, education, marriage), family status (family income, having children or not), disease situation, behavioral risk factors (cigarette smoking, alcohol consumption, and physical activities, diet habits, nutrition supplement consumption, depression, self-care ability, and physical examinations. |
| Zeng, Y., et al. 2015 | China | cohort study | 822 | ≥92 | - | tea | Cognitive Disability | OR (95% CI): [FOXO1A-266: 0.64 (0.38–1.07); FOXO3A-310: 0.59 (0.35–1.00); FOXO3A-292: 0.59 (0.35–1.01)] | gender, age, residence (rural vs urban), education, marital status, regular exercise (yes vs no), smoking (yes vs no), and alcohol drinking (yes vs no). |
| Tomata, Y(a)., et al. 2016 | Japan | cohort study | 13,645 | ≥65 | 5.7 | green tea | Dementia | HR (95% CI): 0.73 (0.61–0.87) | age, sex, history of disease, education level, smoking, alcohol drinking, body mass index, psychological distress score, time spent walking, social support, participation in community activities, motor function score, consumption volume of specific foods, coffee consumption, and energy intake. |
| Tomata, Y(b)., et al. 2016 | Japan | cohort study | 14,402 | ≥ 65 | 5.7 | green tea | Dementia | HR (95% CI): 0.79 (0.70–0.88) | age, sex, history of disease, education level, smoking, alcohol drinking, BMI, psychological distress score (<13, ≥13, missing), time spent walking, motor function score, cognitive function score (0, 1, 2, 3, or missing), number of remaining teeth (0, 1–19, ≥20, missing), energy intake, protein intake. |
| Wang, T., et al. 2017 | China | cross-sectional study | 1,302 | ≥ 60 | - | tea | MCI | OR (95% CI): [60 years-old or more: 0.59 (0.43–0.82); 70 years-old or more: 0.72 (0.49–1.07 )] | —— |
| An, R., et al. 2019 | China | cohort study | 4,749 | ≥80 | 14 | tea | Cognitive Impairment | HR (95% CI): 0.94 (0.81–1.08) | —— |
| Gu, Y. J., et al. 2018 | China | cross-sectional study | 4,579 | ≥ 60 | - | tea | Cognitive Impairment | OR (95% CI): 0.74 (0.57–0.98) | age, sex, BMI, education level, marriage, monthly income, smoking status, alcohol consumption status, dietary, outdoor activities, working status, hypertension, history of diabetes, hyperlipidemia, heart disease, stroke. |
| Fischer, K., et al. 2018 | German | cohort study | 2,622 | ≥ 75 | 10 | green tea | AD and Memory Decline | HR (95% CI): 0.94 (0.86–1.02) | age, gender, BMI, education, APOE ε4 carrier status, smoking status, physical activity score, depression, hypercholesterolemia, and a modified CCI score. |
| Feng, L., et al. 2018 | U.S. | cohort study | 3,844 | ≥65 | 7 | black tea | Cognitive Decline | OR (95% CI): 1.19 (0.81–1.75) | —— |
| Xu, H., et al. 2018 | China | cross-sectional study | 2,131 | ≥60 | - | green tea, black tea, and oolong tea | MCI | OR (95% CI): {Green tea: [men: 0.657 (0.46–0.93); women: 0.82 (0.58–1.16)]; Black tea: [men: 0.738 (0.37–1.49); women: 0.52 (0.24–1.12)]; Oolong tea: [men: 0.390 (0.09–1.68); women: 0.60 (0.13–2.72)]} | age, years of education. |
| Chuang, S. Y., et al. 2019 | China | cross-sectional study | 1,245 | ≥65 | - | tea | Dementia | HR (95% CI): 0.61 (0.44–0.85) | age, sex, education, baseline cognition, body mass index, stroke history, diastolic blood pressure, inflammation status, and stroke occurrence during the study period. |
| Shirai, Y., et al. 2020 | Japan | cohort study | 1,305 | 60–85 | 5.3 | green tea | Cognitive Decline | HR (95% CI): 0.70 (0.52–0.94) | age, sex, survey year at baseline, BMI, smoking status, total physical activity, education years, history of diabetes mellitus, hypertension and hyper lipidaemia, total energy intake, alcohol, green/yellow vegetables, fish, green tea for coffee (and vice versa), and the Mini-Mental State Examination score at baseline. |
| Matsuyama, S., et al. 2020 | Japan | cohort study | 2,923 | ≥65 | 10 | green tea | Incident Functional Disability | HR (95% CI): 0.77 (0.61–0.98) | age, body mass index (BMI), history of disease, current smoker, current drinker, time spent walking, education level, energy, male. |
| Qian, Yu‐Xi, et al. 2020 | China | cross-sectional study | 4,579 | ≥60 | - | tea | Cognitive Impairment | OR (95% CI): 0.66 (0.48–0.90) | —— |
| Wang, Z., et al. 2020 | China | cross-sectional study | 625 | ≥60 | - | tea | MCI | OR (95% CI): 0.285 (0.182–0.447) | —— |
| Huang, W. C., et al. 2021 | China | cross-sectional study | 1,115 | ≥65 | - | tea | Cognitive Frailty and Cognitive Prefrailty | OR (95% CI): [ Prefrailty: 1.56 (1.12–2.18); Frailty: 2.23 (0.75–6.68)] | age, sex, residential area, education level, perceived health status, DDS, body mass index. |

*Abbreviations:* AD, Alzheimer’s disease; BMI: body mass index; CCI, the Charlson comorbidity index; CCM, Critical Care Medicine; CI, confidence interval; CHD, coronary heart disease; CVD, cardiovascular disease; DBP, Astolic blood pressure; DDS: dietary diversity score; DECO, the ‘Détérioration Cognitive Observeé’ (observed cognitive deterioration) questionnaire; HRs, hazard ratios; IADL, the Instrumental Activities of Daily Living; MCI, mild cognitive impairment; MMSE: Mini–Mental State Examination; ORs, odds ratios; PD, Parkinson’s disease; RRs, risk ratios; SBP, Systolic blood pressure; UK, The United Kingdom; U.S., The United States; 3MS, the modified Mini-Mental State.

**Table S2. The quality assessment of cross-sectional.**

| Study | Year | Score |
| --- | --- | --- |
| Cross-sectional (n=13) | | |
| Shinichi Kuriyama | 2006 | 7 |
| Chang-Quan Huang | 2009 | 6 |
| Eha Nurk | 2009 | 5 |
| Marjo H. Eskelinen | 2009 | 8 |
| Ming-Shiang Wu | 2011 | 6 |
| Wei Shen | 2015 | 5 |
| Ying-Jie Gu | 2017 | 6 |
| Tao Wang | 2017 | 6 |
| Hua Xu | 2018 | 6 |
| Shao-Yuan Chuang PhD | 2019 | 8 |
| Yu-Xi Qian | 2020 | 6 |
| Zongqiu Wang | 2020 | 7 |
| Wei-Ching Huang | 2021 | 7 |

AHRQ was used to evaluate the quality of cross-sectional studies.

**Table S3. The quality assessment of cohort and case-control studies.**

| Study | Year | Selection | Comparability | Outcome | Total |
| --- | --- | --- | --- | --- | --- |
| Cohort studies (n=16) | | | | | |
| Qi Dai, MDPhD | 2006 | *** | ** | ** | 7 |
| K. Ritchie | 2007 | *** | ** | *** | 8 |
| Marie-Noël Vercambre | 2009 | *** | ** | *** | 8 |
| Yu-Hui Yao | 2010 | ** | * | ** | 5 |
| Natalia Palacios, Sc.D. | 2012 | **** | ** | ** | 8 |
| Lenore Arab | 2013 | *** | ** | *** | 8 |
| Gang Wang | 2014 | *** | ** | *** | 8 |
| Moeko Noguchi-Shinohara | 2014 | *** | * | ** | 6 |
| Yi Zeng | 2014 | *** | ** | ** | 7 |
| Yasutake Tomata | 2016 | *** | ** | ** | 7 |
| Yasutake Tomata, Ph.D. | 2016 | *** | ** | ** | 7 |
| Lei Feng | 2018 | **** | * | ** | 7 |
| Karina Fischer | 2018 | *** | ** | *** | 8 |
| Yoshiro Shirai | 2019 | *** | ** | *** | 8 |
| Ruopeng An | 2019 | *** | ** | *** | 8 |
| Sanae Matsuyama | 2020 | *** | ** | ** | 7 |
| Case-control studies (n=7) | | | | | |
| D P Forster | 1995 | *** | ** | ** | 7 |
| Harvey Checkoway | 2001 | *** | * | ** | 6 |
| Annlia Paganini-Hill | 2001 | *** | * | ** | 6 |
| Joan Lindsay | 2002 | *** | ** | ** | 7 |
| Keiko Tanaka | 2011 | *** | ** | ** | 7 |
| X. Chen | 2012 | *** | ** | ** | 7 |
| Baoqin Yang | 2014 | *** | ** | ** | 7 |

The NOS scale was used to evaluate the quality of the cohort and case-control studies.


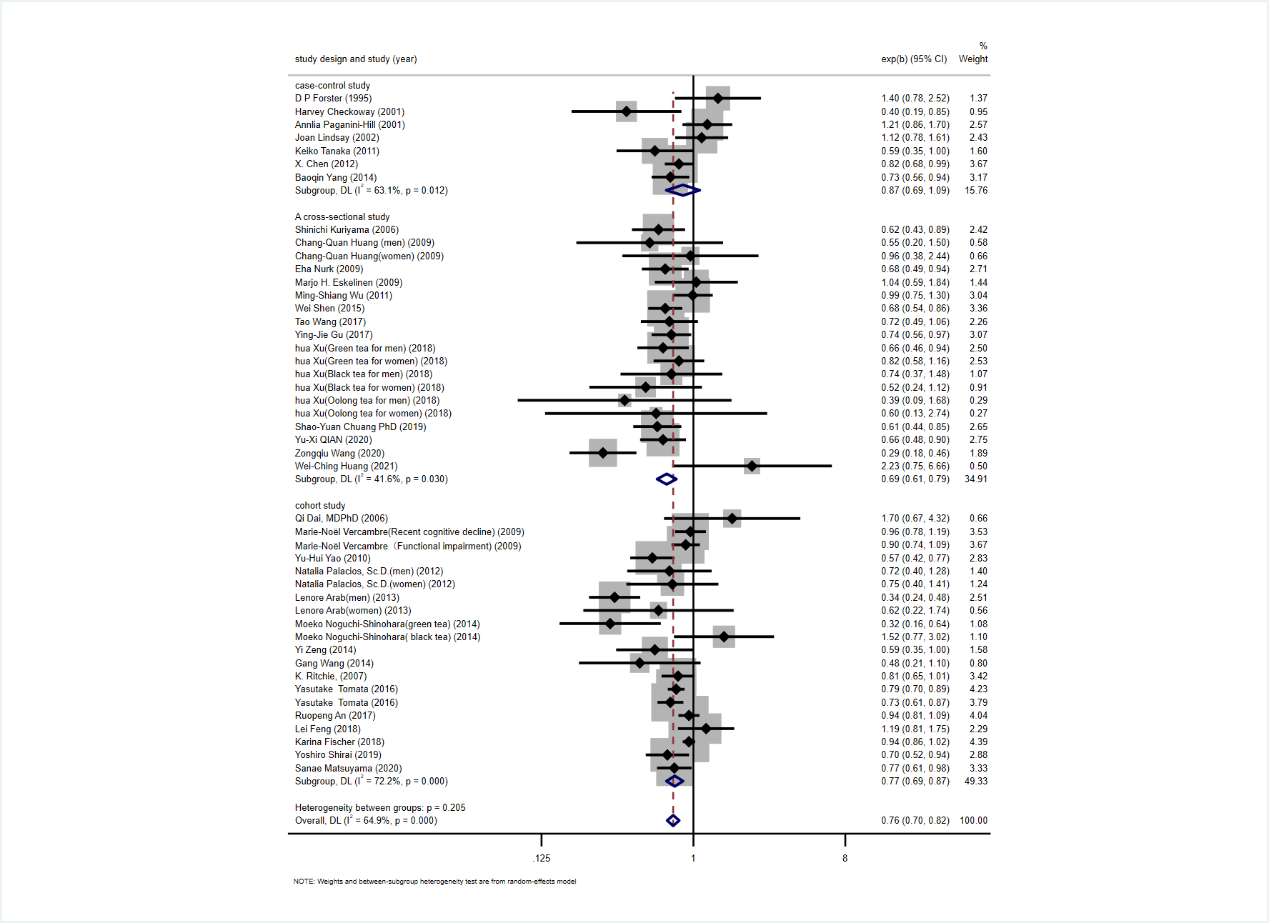


**Figure S1. Subgroup analysis of association between tea drinking** **and cognitive disorders based on study design.**

**Figure S2. Subgroup analysis of association between tea drinking and cognitive disorders based on population.**


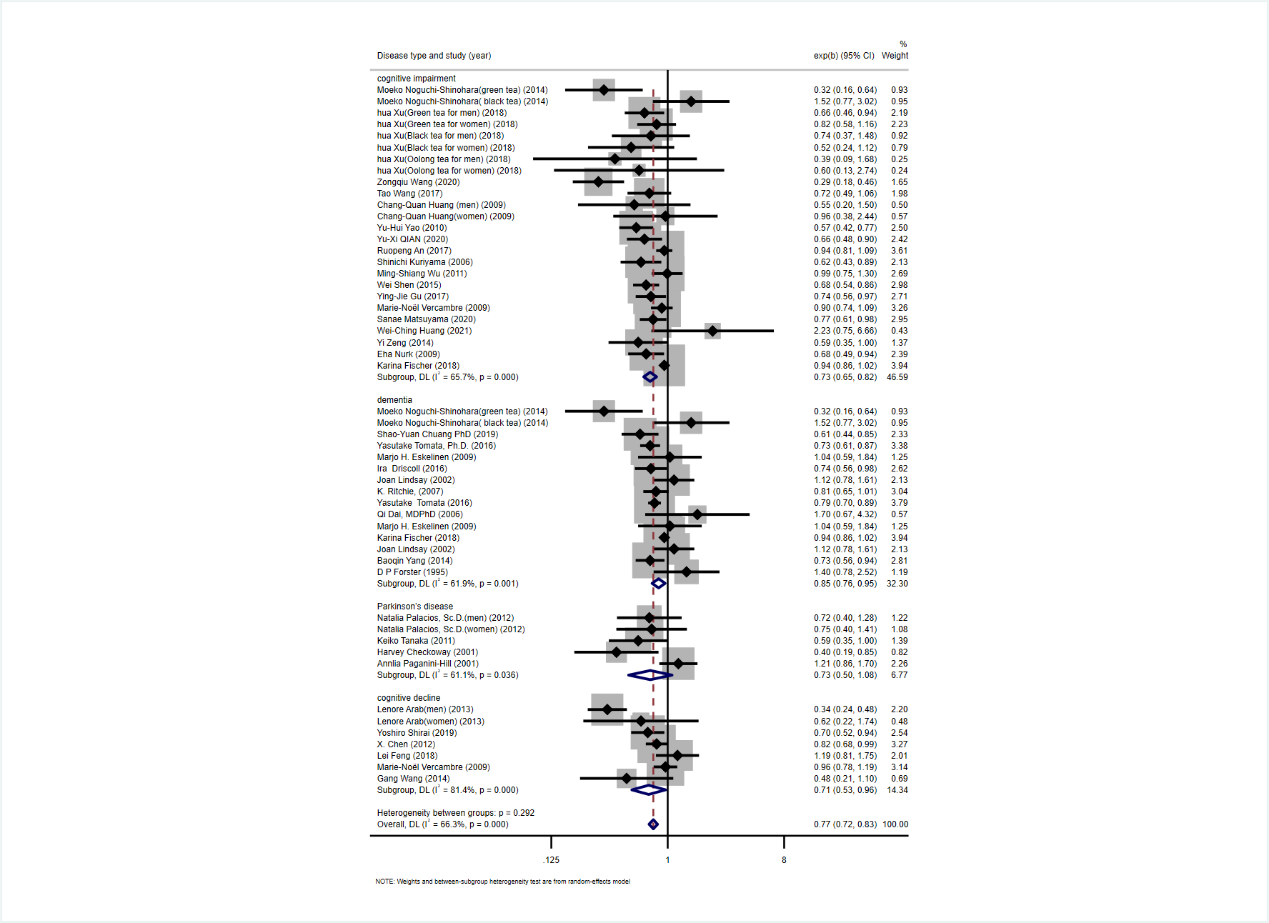


**Figure S3. Subgroup analysis of association between tea drinking and cognitive disorders based on type of cognitive disorders.**


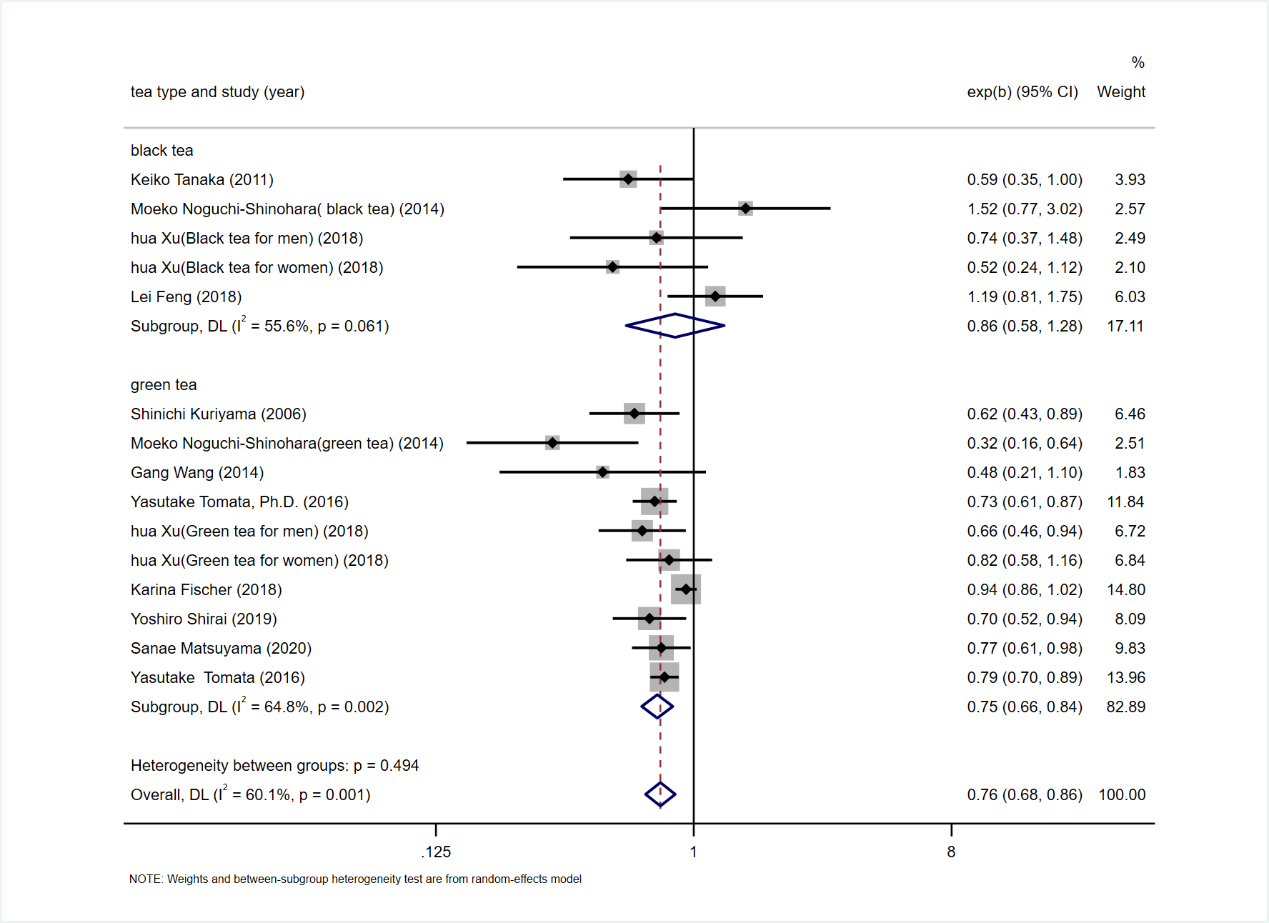


**Figure S4. Subgroup analysis of association between tea drinking and cognitive disorders based on type of tea.**


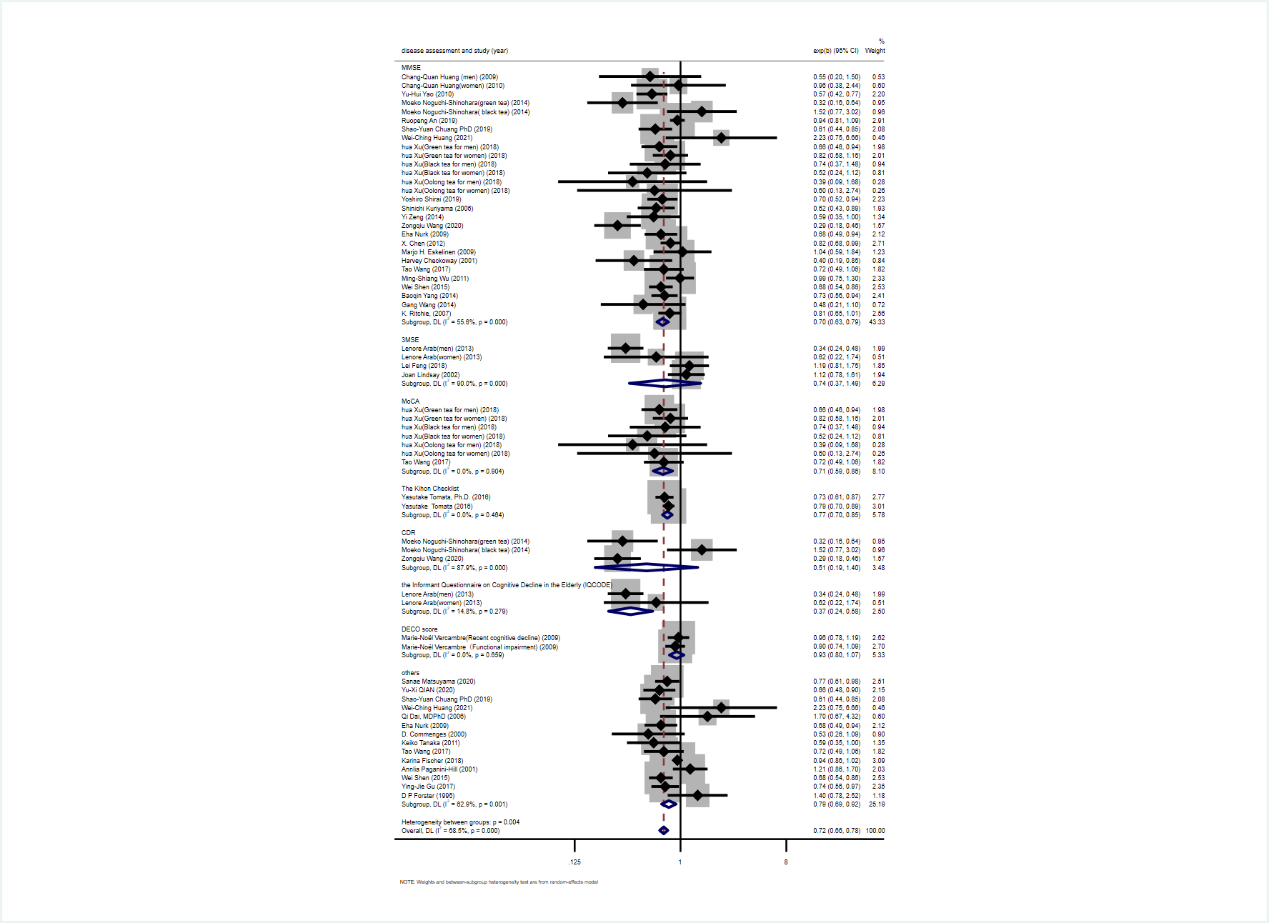


**Figure S5. Subgroup analysis of association between tea drinking and cognitive disorders based on type of outcome assessment.**


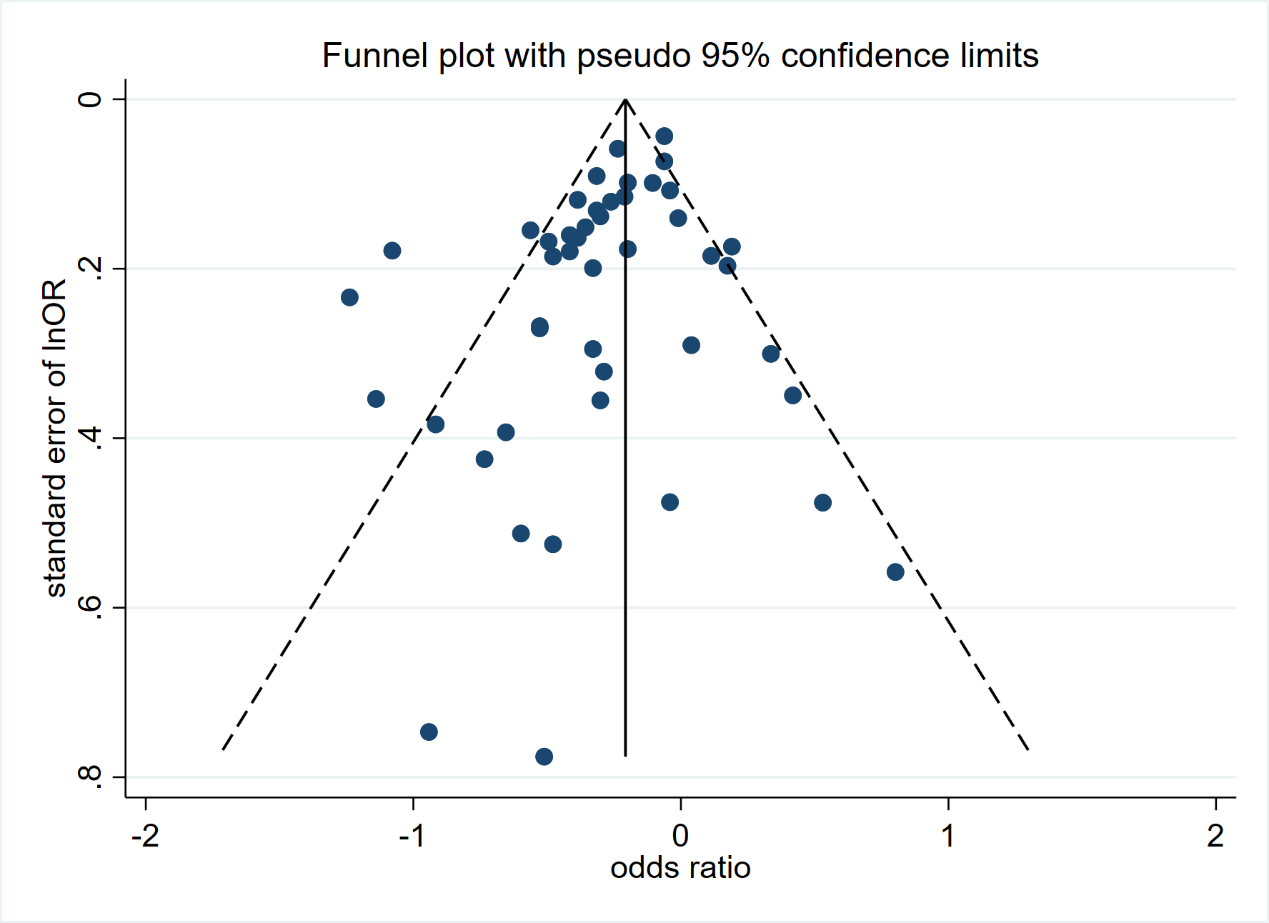


**Figure S6. Funnel plot for assessment of publication bias (If the funnel plot is asymmetric or incomplete, it indicates that there may be publication bias or other bias related to sample size).**
